# Single‐Cell RNA‐seq Reveals a Developmental Hierarchy Super‐Imposed Over Subclonal Evolution in the Cellular Ecosystem of Prostate Cancer

**DOI:** 10.1002/advs.202105530

**Published:** 2022-03-24

**Authors:** Guangzhe Ge, Yang Han, Jianye Zhang, Xinxin Li, Xiaodan Liu, Yanqing Gong, Zhentao Lei, Jie Wang, Weijie Zhu, Yangyang Xu, Yiji Peng, Jianhua Deng, Bao Zhang, Xuesong Li, Liqun Zhou, Huiying He, Weimin Ci

**Affiliations:** ^1^ Key Laboratory of Genomics and Precision Medicine Beijing Institute of Genomics China National Center for Bioinformation Chinese Academy of Sciences Beijing 100101 China; ^2^ Department of Pathology School of Basic Medical Sciences Third Hospital Peking University Health Science Center Beijing 100191 China; ^3^ Department of Urology Peking University First Hospital Beijing 100034 China; ^4^ University of Chinese Academy of Sciences Beijing 100049 China; ^5^ Institute of Urology Peking University Beijing 100034 China; ^6^ National Urological Cancer Center Beijing 100034 China; ^7^ Department of Urology Beijing Aerospace Center Hospital Beijing 100049 China; ^8^ Department of Urology Peking Union Medical College Hospital Beijing 100730 China; ^9^ Institute for Stem cell and Regeneration Chinese Academy of Sciences Beijing 100101 China

**Keywords:** cellular ecosystem, epithelial‐to‐mesenchymal transition, prostate cancer, subtype, transcriptional heterogeneity, tumor subclone

## Abstract

Prostate cancer (PCa) is a complex disease. An ongoing accumulation of mutations results in increased genetic diversity, with the tumor acquiring distinct subclones. However, non‐genetic intra‐tumoral heterogeneity, the cellular differentiation state and the interplay between subclonal evolution and transcriptional heterogeneity are poorly understood. Here, the authors perform single‐cell RNA sequencing from 14 untreated PCa patients. They create an extensive cell atlas of the PCa patients and mapped developmental states onto tumor subclonal evolution. They identify distinct subclones across PCa patients and then stratify tumor cells into four transcriptional subtypes, EMT‐like (subtype 0), luminal A‐like (subtype 1), luminal B/C‐like (subtype 2), and basal‐like (subtype 3). These subtypes are hierarchically organized into stem cell‐like and differentiated status. Strikingly, multiple subclones within a single primary tumor present with distinct combinations of preferential subtypes. In addition, subclones show different communication strengths with other cell types within the tumor ecosystem, which may modulate the distinct transcriptional subtypes of the subclones. Notably, by integrating TCGA data, they discover that both tumor cell transcriptional heterogeneity and cellular ecosystem diversity correlate with features of a poor prognosis. Collectively, their study provides the analysis of subclonal and transcriptional heterogeneity and its implication for patient prognosis.

## Graphical abstract

During tumor evolution, distinct tumor subclones acquire preferential transcriptional subtypes which show different cellular differentiated status. In addition, these subtypes or subclones have close communication with each other or other stromal or immune cells within the tumor ecosystem. These transcriptional heterogeneity and also tumor ecosystem diversity are correlated with patient prognosis.

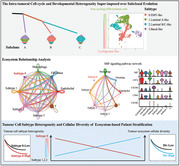

## 1 Introduction

Prostate cancer (PCa) remains the second most frequently diagnosed cancer in men, with more than 1 414 259 new diagnoses and 375 304 deaths per year globally.^[^ ^1^ ^]^ The PCa incidence rate has increased rapidly in China, with an annual percentage change of 12.6% since 2000. A steady increase in advanced or metastatic prostate cancer has been observed at diagnosis in China, advocating for an improvement in treatment strategies.^[^ ^2^ ^]^ Tumors comprise a heterogeneous collection of cells with distinct genetic and phenotypic properties that can differentially promote progression, metastasis, and drug resistance.^[^ ^3^, ^4^, ^5^, ^6^ ^]^ Thus, there is an urgent need to further our understanding of prostate cancer heterogeneity.

Intra‐tumoral heterogeneity arises through both genetic and non‐genetic mechanisms. The stochastic accumulation of mutations through genomic instability results in increased genetic diversity, with the tumor acquiring subclones with distinct genotypes over time.^[^ ^7^ ^]^ Moreover, cancers are hierarchically organized with a stem cell‐like population, sustaining tumor growth through self‐renewal and differentiation.^[^ ^8^ ^]^ The tumor microenvironment also generates intra‐tumoral heterogeneity by exerting different selective pressures in distinct regions of the tumor.^[^ ^9^, ^10^ ^]^ These models are not mutually exclusive and act together to create a complex system with multiple layers of heterogeneity established by the distinct genetic, transcriptomic, and functional properties of different cells.^[^ ^11^, ^12^ ^]^ Subsequently, understanding the function and effect of different cell populations on tumorigenesis, including which features promote tumor initiation, progression, or drug resistance, will also be key.

Emerging single‐cell technologies provide a new opportunity to profile individual cells within tumors and investigate what roles they play in these processes. It is likely that different tumor subclones can acquire transcriptional changes to coordinate subclones’ survival, growth, and competition during tumor evolution. Therefore, it was necessary to evaluate tumor cell transcriptional status among different tumor subclones in untreated PCa patients using single‐cell RNA sequencing technology (scRNA‐seq). Here, we performed scRNA‐seq of primary tumor tissues from 14 untreated PCa patients. On analyses of these data, we observed large degree of intra‐tumoral genomic and transcriptional heterogeneity. Subclones within a patient showed distinct plasticity of cell differentiation states and communication patterns with other cell types within the prostate cancer ecosystem, which has implications for both tumor composition analysis for diagnostics and therapeutic assignment.

## 2 Results

### 2.1 Landscape of the Tumor Ecosystem in Prostate Cancer by Single‐Cell RNA‐seq

**Figure 1:**
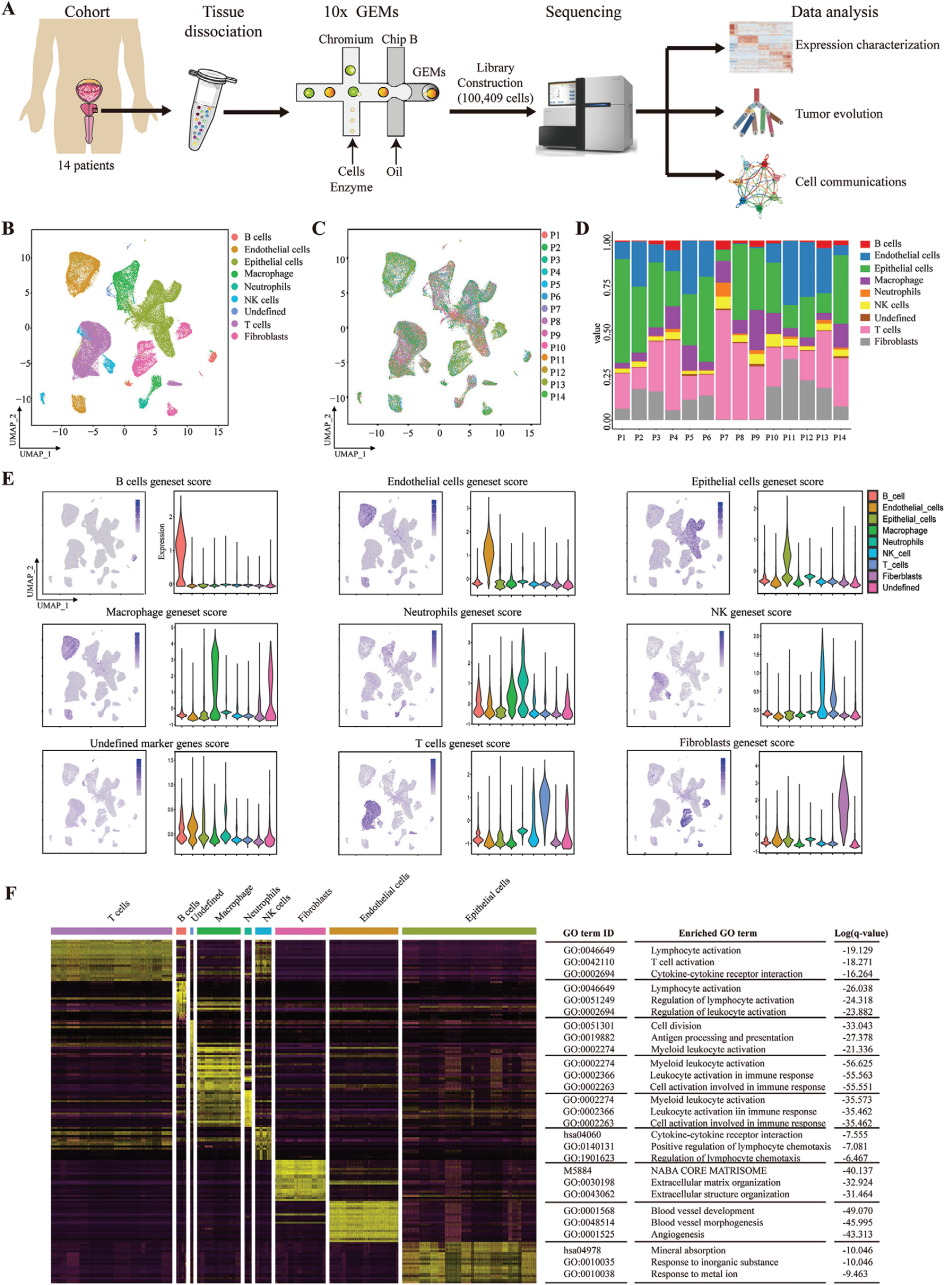
Characterizing the tumor ecosystem in prostate cancer by single‐cell RNA‐seq. A) Overview of the workflow for processing fresh primary prostate cancer samples for scRNA‐seq. B) Uniform manifold approximation and projection (UMAP) visualization of transcriptionally distinct cell populations in the tumor microenvironment from 14 patients. All cells are colored by their cellular identity. C) UMAP of all cells colored by patient. D) Bar graph shows the percentage of each cell type across 14 patients. E) The signature scores were calculated for each cell type in the tumor environment. UMAP maps and violin plots show the expression levels of the signature gene set across 9 clusters. F) Heat map showing the differentially expressed genes that were cell type‐specific in our analysis. The enriched GO terms are shown on the right. *q*‐value < 0.05 was considered statistically significant.

To decipher the landscape of the tumor ecosystem, we generated scRNA‐seq profiles for primary tumors from 14 treatment‐naive prostate cancer patients using a 10× Genomics‐based platform (**Figure** **1**A, and Figure S1A and Table S1, Supporting Information). We obtained a total of 100 409 cells, and 79 596 cells were retained after quality control filtering (Figure S1B–G, Supporting Information). We then performed unsupervised graph‐based clustering and classified different cell clusters into nine major cell types by established marker genes, including those from B cells, endothelial cells, epithelial cells, macrophages, neutrophils, NK cells, T cells, fibroblasts, and undefined cells (Figure 1B,C, and Figures S1H and S1I, Supporting Information). Epithelial cells, endothelial cells, and T cells predominated among the 14 patients (Figure 1D). To further confirm these cell types in the tumor microenvironment, we calculated the aggregated expression score for marker genes in each cell type. UMAP and violin plots show the highly expressed levels of signature genes in corresponding cell types (Figure 1E and Table S2, Supporting Information). Differentially expressed genes were identified for each cell type, and gene ontology analysis showed the enriched GO terms in corresponding cell types (Figure 1F) (e.g., T cell activation in T cells, blood vessel development in endothelial cells, and mineral absorption in epithelial cells). Thus, scRNA‐seq can fully capture the cell diversity in the tumor ecosystem in our cohort.

### 2.2 Tumor Subclones have Preferential Tumor Subtypes in Prostate Cancer

**Figure 2:**
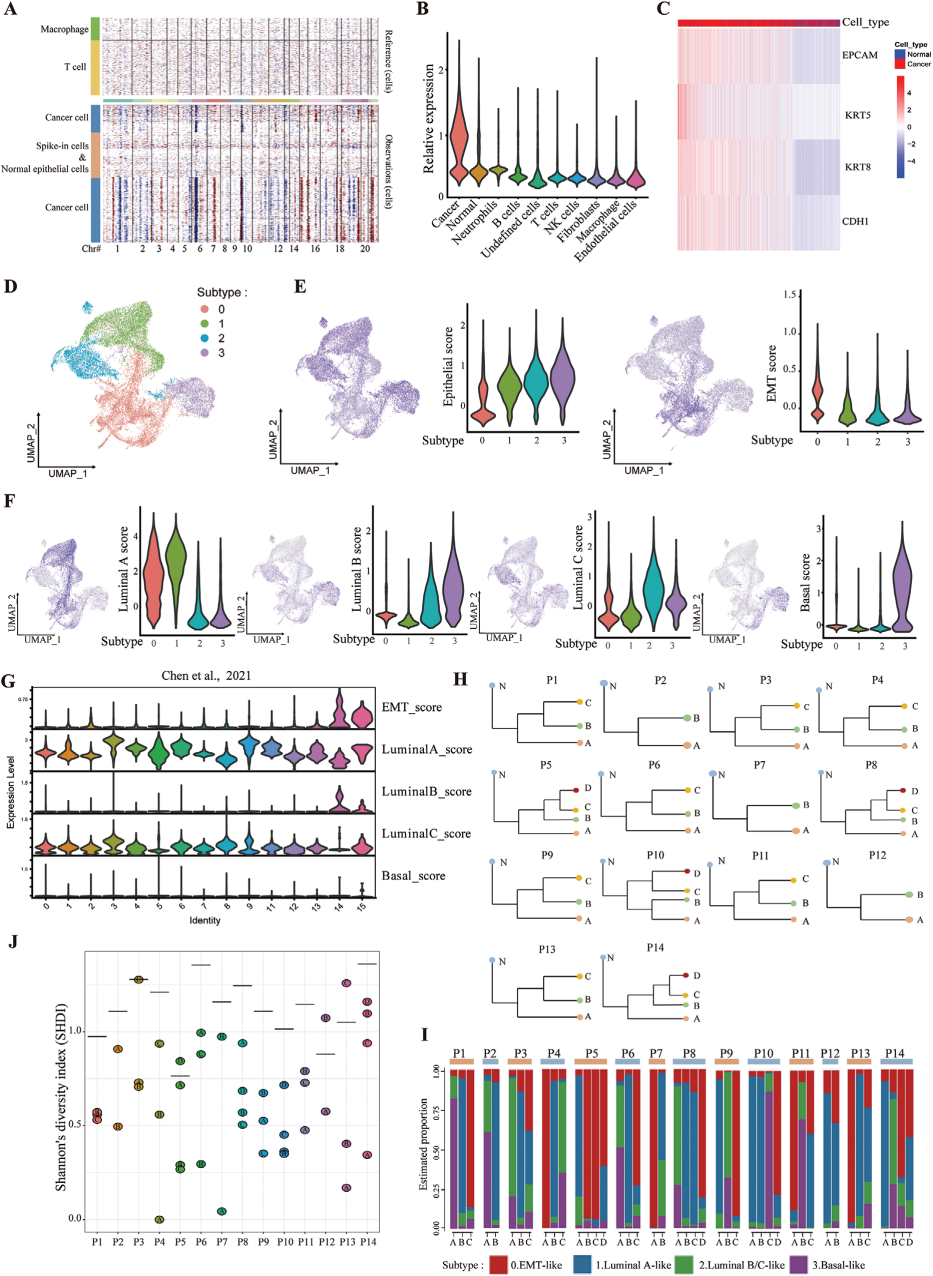
The tumor subclones inferred by inferCNV have preferential tumor subtypes in prostate cancer. A) Inferred large‐scale copy number variations (CNVs) were used to identify cancer (blue) and non‐cancer (brown) cells in a representative patient, P10. Chromosomal regions are shown on the *x* axis. Tumor and normal cells are shown on the *y* axis. B) Violin plot shows the distributions of epithelial gene set scores (average expression of epithelial marker genes, *EPCAM*, *KRT5*, *KRT8*, and *CDH1*) for cells among cancer cells, normal epithelial cells, and other cell types. C) Heat map shows the expression of epithelial marker genes across tumor and normal epithelial cells (columns), sorted by epithelial gene set scores. D) UMAP visualization of tumor cell subtypes across 14 patients by unsupervised clustering. E) The epithelial and EMT geneset score were calculated and visualized in 4 tumor subtypes. F) UMAP and violin plots show the expression levels of genes in the signature gene set (luminal A, luminal B, luminal C, and basal cell markers) across 4 tumor subtypes. G) The according signature geneset scores were calculated from a published dataset by Chen et al. H) Tumor subclone evolutionary trees were inferred from the copy number for 14 patients using inferCNV. N, normal cells. I) Bar graph shows the percentage of each tumor subtype across different tumor subclones in 14 patients. J) Shannon's diversity index (SHDI) was calculated for each tumor subclone in 14 patients to assess the preference of tumor subtypes. Low SHDI indicated the predominance of specific tumor subtype in respective tumor subclones. The horizontal bar in each patient showed the SHDI with total subtypes distribution in that patient regardless of tumor subclones.

Next, we sought to investigate the genomic and transcriptional heterogeneity in prostate cancer cells. We first distinguished 20 681 malignant cells and 2802 normal prostate epithelial cells based on large‐scale chromosomal copy number variations (CNVs) in each cell inferred by inferCNV (**Figure** **2**A and Figure S2A, Supporting Information). Then, we confirmed this classification by calculating an epithelial score with epithelial marker genes, *EPCAM*, *KRT5*, *KRT8*, and *CDH1* (Figure 2B). The epithelial score in cancer cells was much higher than that in normal prostate epithelial cells or other stromal cells, which reflected the accuracy of our approach (Figure 2C). Then, we performed an analysis of transcriptional heterogeneity within tumor cells. Unsupervised clustering revealed 4 different tumor subtypes (0, 1, 2, and 3) in all tumor cells across 14 patients (Figure 2D, and Figure S2B and Table S3, Supporting Information). The aggregated expression score for marker genes in epithelial cells, EMT (epithelial‐to‐mesenchymal transition), luminal A/B/C, and basal cells was analyzed for all subtypes. Subtype 0 had a much lower epithelial score and a much higher EMT score, indicating an “EMT‐like” phenotype (Figure 2E). Subtype 1 had a high signal for “luminal A‐like” cells, subtype 2 had a high signal for both “luminal B‐like” and “luminal C‐like” cells, and subtype 3 had a high signal for “basal‐like” cells (Figure 2F). To confirm our findings, we analyzed the tumor cells from published PCa scRNA‐seq dataset^[^ ^12^ ^]^ (Figure 2G). We found that 14# and 15# have high expression of EMT signature genes corresponding to our subtype 0. Other tumor cells highly expressed the luminal A and C signature genes although not the luminal B and basal genes. In addition, we also re‐clustered the tumor cells with more subpopulations. The new subpopulations were consistent with our defined 4 tumor subtypes (Figure S2C–E, Supporting Information). Thus, we used these 4 tumor subtypes to the following analysis. Next, we inferred the tumor subclonal structure in each patient based on the CNV profiles from inferCNV results (Figure 2H). We aimed to determine whether a specific tumor subclone in one patient tended to have a specific tumor subtype. The results suggested that almost all patients had predominately one or two tumor subtypes within their tumor subclones (Figure 2I,J). These results indicated that during tumor subclonal evolution preferential orientation of transcriptional heterogeneity occurred in different tumor subclones.

### 2.3 The Intra‐Tumoral Cell Cycle and Developmental Heterogeneity Within Different Tumor Subtypes and Subclones

**Figure 3:**
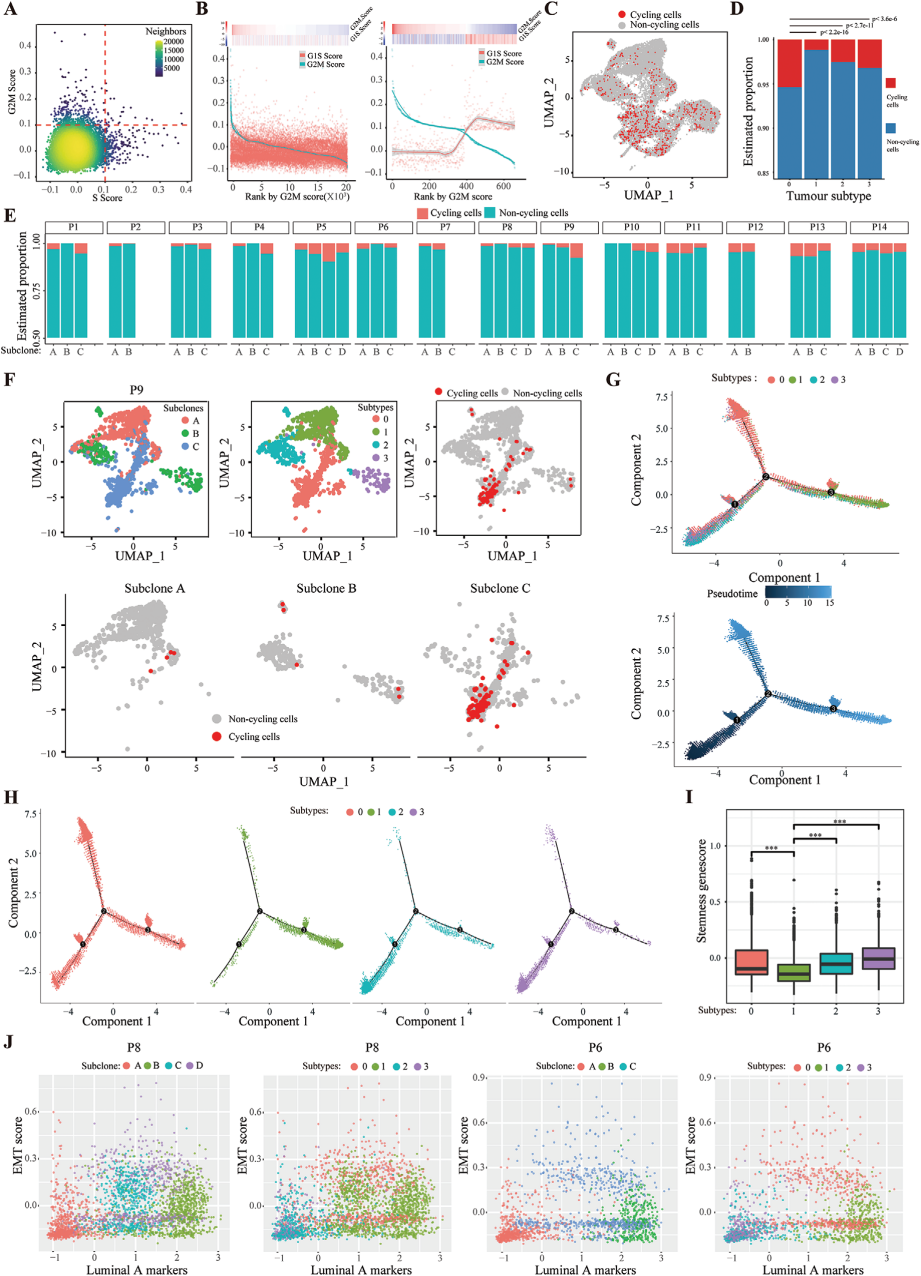
Heterogeneity of the cell cycle and differentiation architecture among different tumor subtypes and subclones. A) Classification of tumor cells into cycling and non‐cycling cells based on the relative expression of gene sets associated with G1/S (*x* axis) and G2/M (*y* axis). B) A subset of cycling cells with high expression of genes related to G1/S and G2/M. Shown are the average expression levels of the G1/S and G2/M gene sets in all cells (left) or among the putative cycling cells (right) ordered by decreasing expression of the G2M genes. C) UMAP plot shows the cycling and non‐cycling cells among tumor cells. D) Bar graph shows the percentages of cycling and non‐cycling cells across different tumor subtypes in 14 patients. *p* value was calculated by chi‐squared test. E) Bar graph shows the percentages of cycling and non‐cycling cells across different subclones within each patient. F) UMAP plot shows tumor subtypes, subclones, and cycling cells in one representative patient, P9. (G) and (H) Monocle analysis of 4 tumor subtypes. Cells were ordered by pseudotime. I) The stemness geneset score were calculated for each tumor subtype. *p* value was calculated by two‐tailed *t*‐test, ***p < 0.001. J) The intra‐tumoral developmental heterogeneity super‐imposed over subclonal evolution.

Intra‐tumoral heterogeneity that has been observed among malignant glioma cells^[^ ^13^ ^]^ can reflect the cell cycle program, as actively cycling cells upregulate a large number of associated genes. We first assessed the cell proliferation status using cell cycle‐associated G1/S and G2/M gene sets (**Figure** **3**A, and Figures S3A and S3B, Supporting Information). We found that only a small proportion of tumor cells proliferated, which is consistent with clinical observations in a published paper^[^ ^12^ ^]^ (Figure 3B). Then, we assessed the proliferation fractions in tumor subtypes and subclones. The results suggested that subtype 0, had a higher proliferation rate than the other 3 subtypes (Figure 3C,D). We also found great heterogeneity in the cell proliferation status among different subclones and different patients (Figure 3E). The subclones with preferential subtype 0 phenotype had many more proliferating cells than other subclones within one representative patient (Figure 3F).

Moreover, other common patterns of variability are primarily correlated with epithelial developmental cell types, such as basal and luminal cells. Thus, we used Monocle to perform differentiation trajectory analysis in cancer cells. The pseudotime results showed that developmental hierarchy started with subtype 2 and 3 and progressing towards subtype 1 (Figure 3G). These results indicated that tumor cells in subtype 2 and 3 have a much lower differentiation status compared with those in subtype 1, which showed the high similarities with luminal A differentiated epithelial cells. Indeed, differential gene expression analysis attributed the three co‐expressed gene clusters to the three subtypes 1, 2, and 3, concordant with the pseudotime states (Figures S3C and S3D, Supporting Information). Strikingly, subtype 0, EMT‐like cells, distributed along different axes of the pseudotime plot, which have a spectrum of transitions states along the epithelial–mesenchymal axis (Figure 3H). Consistently, pathway analysis by gene set variation analysis (GSVA) revealed that the EMT, TGF*β*, and hypoxia pathways were enriched in cells from subtype 0 (Figure S4A, Supporting Information). Furthermore, GSVA analysis also identified shared enriched pathways between subtype 0 and other subtypes (Figure S4A, Supporting Information). Strikingly, in recent years, an increasing number of terms have accrued to describe cell plasticity along the epithelial–mesenchymal axis, such as partial EMT^[^ ^14^ ^]^ and hybrid E/M^[^ ^15^ ^]^ states for the ability of cells to switch freely between these various states with enhancing stemness and metastatic capacity.^[^ ^16^ ^]^ These results are consistent with the observations that subtype 1 has a lower stemness genescore whereas subtype 0, 2, and 3 have a higher stemness genescore (Figure 3I). Consistent with the above mentioned results (Figure 2H,I), during tumor evolution, preferential orientation of transcriptional heterogeneity occurred in different tumor subclones (Figure 3J). Collectively, these results indicate that tumor cells acquire cell cycle and developmental heterogeneity during subclonal evolution.

### 2.4 Expression Program Varys among Heterogeneous Developmental Subtypes

**Figure 4:**
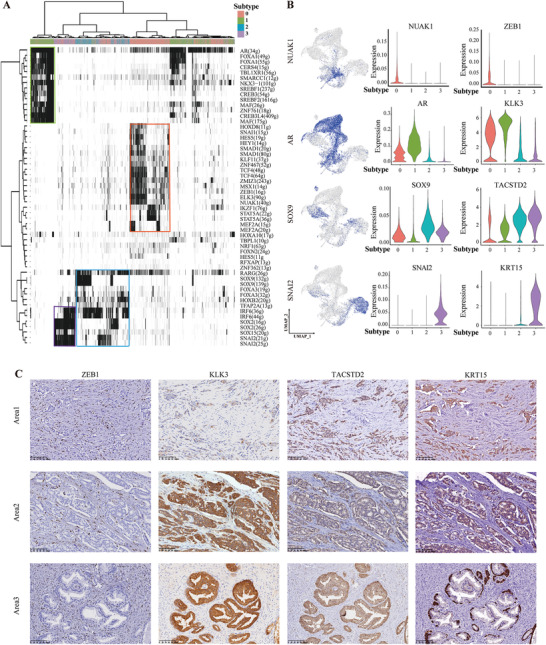
Expression programs vary among the heterogeneous developmental subtypes. A) The heat map shows the binary activities of the TF regulons underlying different tumor subtypes. B) UMAP plots show binary activities of the TF regulons in all tumor cells (left panel). Violin plot shows the distributions of activities of the TF regulons (middle panel) and targeted marker genes across 4 tumor subtypes (right panel). C) Representative IHC images of ZEB1, KLK3, TACSTD2, and KRT15 in the serial tissue sections of a representative patient, P3. Area 1 and 2 are the regions of tumor cells, area 3 is a region of normal adjacent tissue.

To explore the potential molecular basis driving the distinct developmental subtypes, we next used single‐cell regulatory network inference and clustering (SCENIC) to identify the underlying regulatory network in each tumor subtype. As expected each tumor subtype was driven by relevant TFs,^[^ ^17^ ^]^ including NUAK1, SNAI1, and ZEB1 for the EMT‐like subtype 0 cells; AR, FOXA1 for the differentiated luminal A‐like subtype 1 cells; SOX9, IRF6 for the luminal B/C‐like luminal progenitor subtype 2 cells; and SOX2, SNAI2 for the basal‐like subtype 3 cells (**Figure** **4**A). Consistently, up‐regulating the known markers of each corresponding subtype by relevant TFs as key regulators were also identified in each subtype, including ZEB1 by NUAK1 for subtype 0; KLK3 by AR for subtype 1; TACSTD2 by SOX9 for subtype 2; and KRT15 by SNAI2 for subtype 3 (Figure 4B). Moreover, the expression of representative markers and relevant TFs for each subtype were also significantly correlated in the TCGA PCa cohort (Figure S4B, Supporting Information). More importantly, the heterogeneous developmental subtypes of tumor cells were also confirmed by immunohistochemistry assay in sections from patients’ samples. We detected tumor areas expressing EMT marker, tumor areas expressing epithelial markers for each subtype (Figure 4C). Thus, these analyses showed a heterogeneous expression program within different developmental subtypes in PCa.

### 2.5 The Cell Plasticity in Malignant Cells May Be Associated with Tumor Microenvironmental Cues

**Figure 5:**
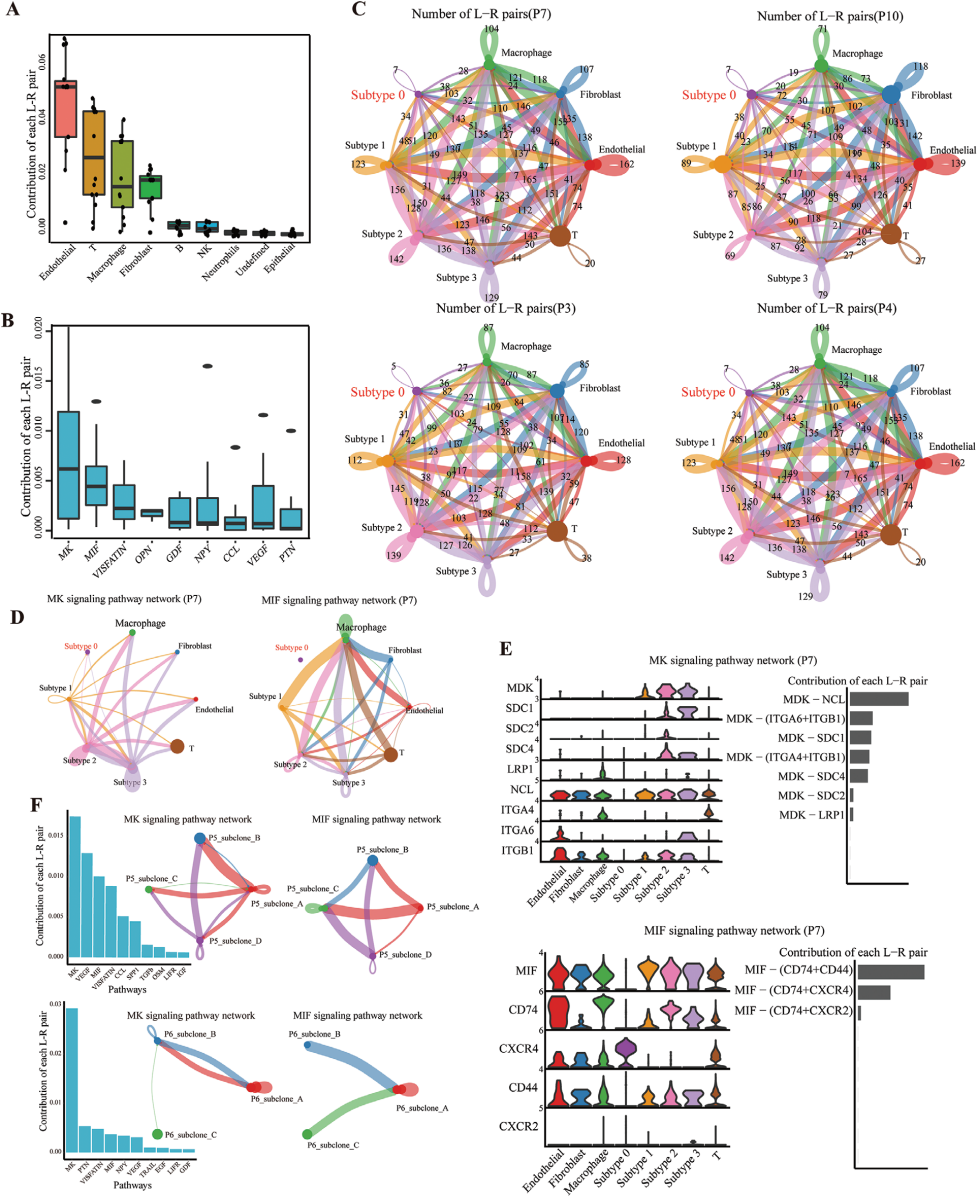
Differential strengths of cell communication exist within different tumor subtypes/subclones and cell types in the tumor microenvironment. A,B) The difference in cell communication strength between tumor cells and other normal cell types was calculated among signalling pathways in all 14 patients. MIF, macrophage migration inhibitory factor. C) Number of significant ligand‐receptor pairs between any pair of two cell populations. The edge width is proportional to the indicated number of ligand‐receptor pairs. Circle sizes are proportional to the number of cells in each cell group and edge width represents the communication probability. D) The inferred MK and MIF signaling networks in one representative patient, P7. E) Expression distribution of MK and MIF signaling genes and relative contribution of each ligand‐receptor pair to the overall MIF signaling network in P7. F) The difference in cell communication strength between different subclones was calculated among signalling pathways in P5 and P6. The signaling networks were shown within subclones from P5 and P6 in MK and MIF pathways.

Microenvironmental cues such as soluble factors (growth factors and cytokines) or intercellular communication network may drive the cell plasticity of subclones or subtypes. Thus, we applied CellChat,^[^ ^18^ ^]^ a tool that is able to quantitatively infer and analyze intercellular communication networks among ligands, receptors, and their cofactors from scRNA‐seq data. We found that endothelial cells, T cells, macrophages, and fibroblasts were the dominant communication hubs (**Figure** **5**A). Moreover, the MK (Midkine), MIF (macrophage migration inhibitory factor), and VISFATIN/nicotinamide phosphoribosyltransferase (NAMPT) signalling pathways were the pathways with the most interactions (Figure 5B).

To evaluate whether intra‐tumoral subclones shaping their developmental subtypes through communications with other spatially colocalized cell populations in tumor ecosystem, tumor cells were separately evaluated as subclones or subtypes. Strikingly, the subclone with dominant subtype 0 phenotype had weaker communication strength both within tumor cells and with other cell populations (Figures S5A and S5B, Supporting Information). These results were consistent with the knowledge that the plasticity of EMT including the loss of strong cell–cell contacts, cell polarity, and immobility. Since the proportion of tumor cell with subtype 0 phenotype showed inter‐tumor heterogeneity, we next validated the generality of this observation in all other patients. As shown in Figure 5C, we confirmed the finding in four representative patients with varying amount of subtype 0 tumor cells. Then, we investigated the communication strength between different subtypes and other stromal cell types among MK and MIF signalling pathways which are activated in PCa patients.^[^ ^19^, ^20^ ^]^ We found clearly different communication strength among these cells (Figure 5D,E and Figure S5C, Supporting Information). We further investigated the communication strength within tumor subclones and also found the significant difference in MK and MIF signalling pathways (Figure 5F and Figure S5D, Supporting Information). Collectively, these results indicated that the different stromal cell types can differentially interact with different tumor subtypes/subclones in patients and may thus explain different tumor behaviors.

### 2.6 Both Tumor Cell Transcriptomic Heterogeneity and Cellular Ecosystem Diversity Correlate with Features of a Poor Prognosis

Given that the role of tumor microenvironmental cues in shaping tumor cell plasticity, we next evaluated whether both intra‐tumoral cellular ecosystem heterogeneity and tumor cell transcriptomic heterogeneity may drive disease progression. We established two computational scores to quantify different aspects of tumor heterogeneity. Tumor cell transcriptomic heterogeneity describes the composition of tumor cell transcriptional subtypes within a tumor. Cellular ecosystem diversity quantifies the diversity of co‐existing tumor cells and other cell types within the tumor ecosystem.

**Figure 6:**
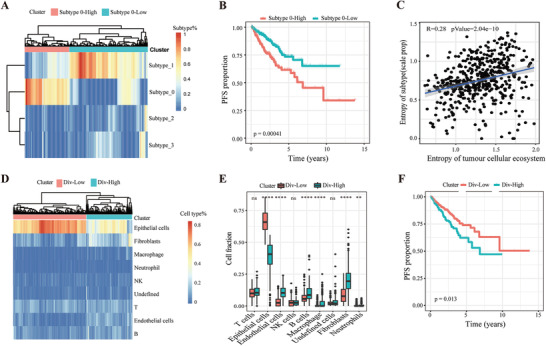
Both tumor cell transcriptomic heterogeneity and cellular ecosystem diversity predict patient prognosis in the TCGA cohort. A) Unsupervised clustering reveals two clusters, Subtype 0‐High and Subtype 0‐Low, based on the fractions of intra‐tumoral subtypes deconvoluted by CIBERSORTx in the TCGA cohort of prostate cancer patients. B) Kaplan–Meier plot shows that Subtype 0‐High had shorter progression‐free survival (PFS) than Subtype 0‐Low within the TCGA dataset. *p* value was calculated by log‐rank test. C) Pearson correlation coefficient of tumor cell transcriptional heterogeneity and cellular ecosystem diversity. Shannon's diversity index (SHDI) is used to evaluate heterogeneity. D) Unsupervised clustering reveals two clusters, Div‐High and Div‐Low, based on the cellular ecosystem deconvoluted by CIBERSORTx in the TCGA cohort of prostate cancer patients. E) The differences in cell content among different cell types in the tumor microenvironment were calculated between Div‐High and Div‐Low in the TCGA cohort. *p* value was calculated by two‐tailed *t*‐test, *p < 0.05; **p < 0.01; ***p < 0.001; NS, not significant. F) Kaplan–Meier plot shows that the Div‐High group had shorter progression‐free survival (PFS) than the Div‐Low group within the TCGA dataset. *p* value was calculated by log‐rank test.

First, we used CIBERSORTx to deconvolute the compositions of 4 tumor transcriptional subtypes in the TCGA cohort based on our scRNA‐seq datasets. We calculated the fraction of each subtype in prostate cancer patients from the TCGA dataset. Unsupervised clustering analysis revealed two clusters (**Figure** **6**A). Cluster 1 had a much higher proportion of subtype 0 malignant cells and cluster 2 had a much higher proportion of subtypes 1, 2, and 3. The progression‐free survival of the patients in cluster 1 was significantly poorer than that of patients in cluster 2, which indicates that transcriptional subtype 0 was associated with a worse prognosis (Figure 6B and Figure S6A, Supporting Information).

Considering the extensive interaction among different cell types in the tumor microenvironment, we hypothesized that tumor cellular ecosystem diversity may affect the heterogeneity of tumor subtypes. Thus, we calculated Shannon's diversity index (SHDI) to assess the correlation of tumor subtype heterogeneity and cellular diversity of the tumor ecosystem. We found a positive correlation (according to Shannon's diversity index [SHDI]) between tumor subtype heterogeneity and cellular diversity in the tumor ecosystem (Figure 6C and Figure S6B, Supporting Information). This result indicated that the cellular complexity of the tumor ecosystem may shape the components of tumor subtypes through cell communication and thus affect patient survival.

Moreover, we speculated that the tumor ecosystem may also have some underlying patterns to govern patient survival. Therefore, we performed unsupervised clustering analysis using CIBERSORTx‐inferred cellular diversity in the tumor ecosystem and identified two clusters, Div‐High and Div‐Low. Div‐High had greater cellular diversity than Div‐Low (Figure 6D). Furthermore, compared to the Div‐Low group, the Div‐High group had significantly fewer epithelial cells but significantly more endothelial cells, B cells, macrophages, fibroblasts, and neutrophils (Figure 6E). Strikingly, the progression‐free survival of the patients in the Div‐High group was significantly poorer than that of the patients in the Div‐Low group, which indicates that higher tumor cellular ecosystem diversity was also associated with a worse prognosis in the TCGA dataset (Figure 6F). Consistent with these findings, a poor outcome was also predicted by the lower proportion of epithelial cells and the higher proportions of endothelial cells, macrophages, neutrophils, and undefined cells (Figure S6C–H, Supporting Information). The proportion of other cell types within the tumor ecosystem was not associated with the clinical outcome (Figure S6I–L, Supporting Information). Overall, both higher proportion of subtype 0 tumor cells and the higher cellular diversity of the tumor ecosystem were associated with a poorer outcome.

## 3 Discussion

PCa is a clinically heterogeneous disease. Many attempts have been made to unravel intra‐tumoral genetic heterogeneity in PCa.^[^ ^6^, ^21^, ^22^ ^]^ However, it became clear that non‐genetic transcriptional heterogeneity may also play important role in cancer evolution.^[^ ^12^, ^23^, ^24^ ^]^ Therefore, understanding how those tightly interconnected genetic and non‐genetic mechanisms lead to the establishment of evolving heterogeneous PCa is of clinical significance. In addition to cell proliferation states and expression programs, copy number aberrations can be inferred from scRNA‐seq data. scRNA‐seq analysis provides a viable strategy to map developmental states onto tumor subclonal evolutions, which may help to understand collective behavior and regulatory mechanisms within a tumor ecosystem.^[^ ^13^ ^]^ In this study, we comprehensively characterized the intra‐tumoral heterogeneity in 14 untreated prostate cancer patients by scRNA‐seq technology. We identified genetic evolving subclones of tumor cells by inferCNV in all patients. Strikingly, our analysis revealed four transcriptional subtypes, EMT‐like (subtype 0), luminal A‐like differentiated cells (subtype 1), luminal B/C progenitor‐like cells (subtype 2), and basal‐like cells (subtype 3). Every tumor contained malignant cells corresponding to four transcriptional states at various extents. We confirmed our “EMT‐like” and “Luminal A‐ and B/C‐like” subtypes using scRNA‐seq dataset from a recently published paper,^[^ ^12^ ^]^ although they didn't find the “basal‐like” subtype in their dataset. This may be due to the different patient cohort because we indeed identified the basal gene highly‐expressed tumor cells in prostate cancer tissue. In addition, we found that genetic distinct subclones presented with preferential developmental subtypes. This phenomenon is consistent with a previous study in human oligodendroglioma which CNV subclones differed in relative distribution of different tumor cell subpopulations.^[^ ^25^ ^]^ These results suggested that non‐genetic developmental heterogeneity may super‐impose over subclonal evolution in PCa. Thus, we provided evidences at single‐cell level that PCa tumor cells displayed significant non‐genetic plasticity, supporting that transcriptional heterogeneity may also play important role in cancer progression, adaptation, and evolution within tumor ecosystem.

Further supporting this notion, subtype 0 cells showed the plasticity along the epithelial–mesenchymal axis. It has been showed that cells moving forward and reversibly on the epithelial–mesenchymal axis, enabling them to adapt to and appropriate new environments during tumor progression.^[^ ^26^, ^27^ ^]^ Indeed, the subclone with dominant subtype 0 phenotype had weaker communication strength both within tumor cells and with other cell populations, suggesting loss of strong cell–cell contacts enabling subclones gaining mesenchymal phenotype to adapt to changing microenvironment. Furthermore, CellChat analysis enables the identification of key intercellular communication pathways, such as MK and MIF signalling pathways in PCa patients. Notably, these highly communicated signalling pathways have been reported to be activated as novel biomarkers and therapeutic targets in PCa.^[^ ^19^, ^20^, ^28^ ^]^ This result suggests that scRNA sequencing approaches could indeed be used to address the phenotypic heterogeneity of cancer cell populations and may be of crucial importance in designing personalized therapeutic approaches.

**Figure 7:**
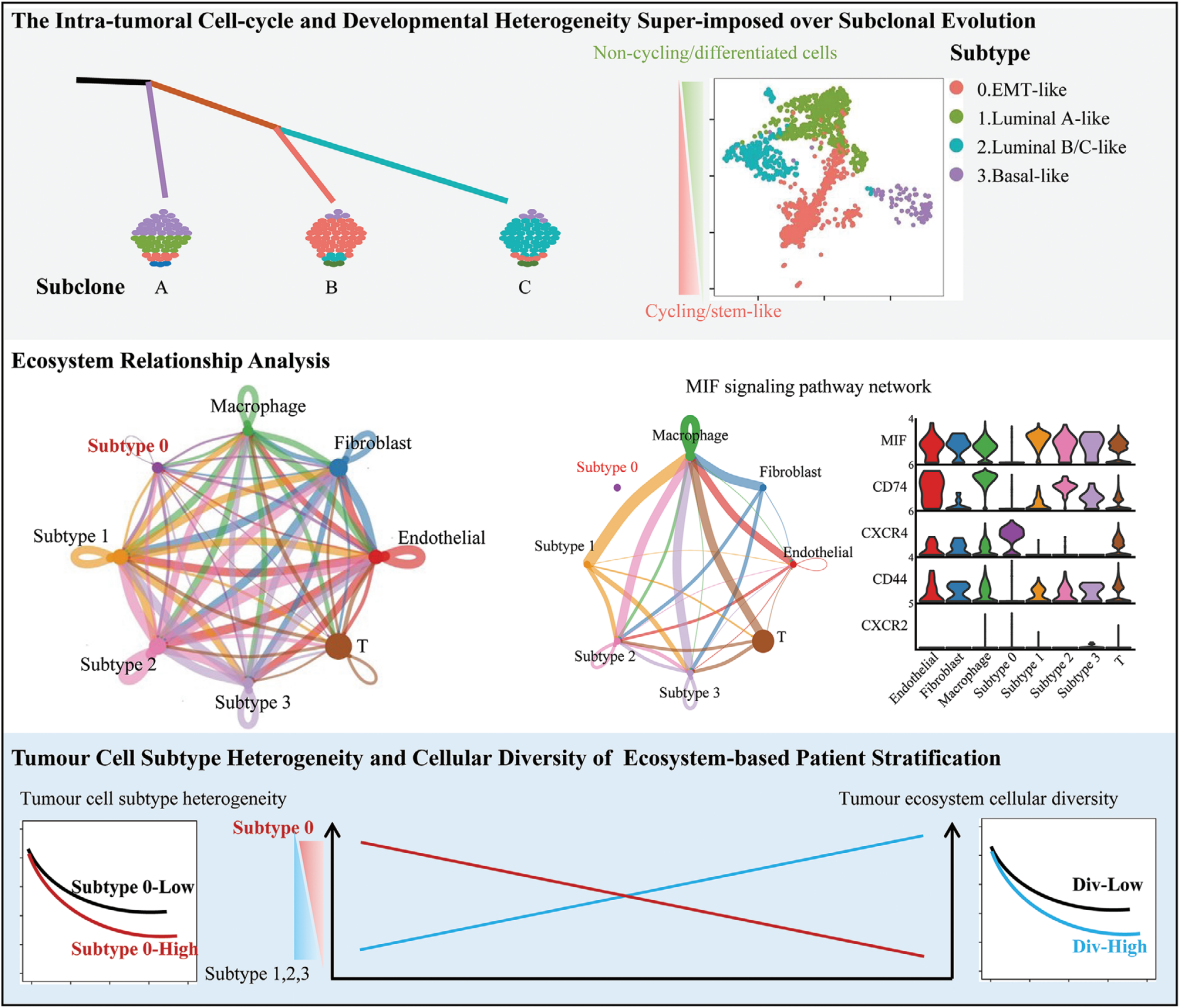
Model of genetic subclone evolution coupled with developmental hierarchy in the tumor ecosystem.

Moreover, it was reported that cells in an intermediate state along the epithelial–mesenchymal axis are plastic, invasive, and highly metastatic.^[^ ^29^ ^]^ Whereas with limited number of cases and insufficient follow‐up period in current cohort, we further validated the role of tumor cell transcriptional plasticity in patients’ outcome in TCGA cohorts. Given that the role of tumor microenvironmental cues in shaping tumor cell plasticity, indeed we identified both a higher proportion of subtype 0 tumor cells and the higher cellular diversity of the tumor ecosystem were associated with a poorer outcome (**Figure** **7**). Limitations of the current study include the low sample size to produce conclusive evidence of intercellular communication for the tumor phenotype. Further study combing high‐resolution spatial scRNA‐seq and genetic manipulation of genes and pathways of interest will precisely map key regulatory nodes in shaping tumor phenotype and facilitate the development of effective therapies to successfully induce non‐genetic cellular differentiation.

The present study demonstrated that non‐genetic transcriptional heterogeneity existed in PCa patients and during tumor evolution, distinct subclones acquired preferential transcriptional subtypes. These transcriptional subtypes hierarchically organized into stem cell‐like and differentiated status and one subtype presented with transitions states along the epithelial–mesenchymal axis. In addition, subtypes/subclones showed different communication strengths with other stromal cell types within the tumor ecosystem. Finally, patients with higher proportion of “EMT‐like” subtype or higher cellular diversity of tumor ecosystem were correlated with a poorer outcome.

## 4 Experimental Section

### Sample Collection and Dissection

A total of 14 surgically resected tissue samples were collected from patients with prostate cancer, and the pathological type was prostatic acinar adenocarcinoma. No endocrine therapy was conducted on any patients before surgery. The tissue samples were digested into single‐cell suspensions and then cell capture and cDNA synthesis were carried out on a 10× Genomics platform. Transcriptome sequencing was performed on a NovaSeq 6000 platform after meeting all quality controls. The study was approved by the Peking University Third Hospital Medical Science Research Ethics Committee (No. IRB00006761‐M2021324).

### Processing Single‐Cell RNA‐seq Data

Raw data generated with the 10× Genomics platform were aligned to the GRCh38 reference genome using Cell Ranger software (v4.0.2)^[^ ^30^ ^]^ to obtain the UMI matrix, which was further imported into R (v4.0.3) and processed with the Seurat package (v3.2.1). Cells with a detected gene number <200 or >5000 or a high mitochondrial transcript ratio (>15%) were excluded. After normalization and scaling, the batch effect between patients was then removed using the mutual nearest neighbor algorithm in Seurat. The batch‐corrected matrix was used for further analysis and visualization. The top 2000 highly variable genes were extracted to perform principal component analysis (PCA), and the top 30 principal components (PCs) were used for cluster analysis. Cell type were annotated by the SingleR package (v1.2.4) and then checked manually.

### Identification of Marker Genes and Enrichment Analysis

To identify differentially expressed marker genes for each cell type, the FindAllMarkers function in Seurat was used under default parameters. Marker genes were selected as those with adjusted *p* values less than 0.05, average logFC larger than 0.25, and percentage of cells with expression higher than 0.25. Gene enrichment analysis was performed for marker genes of each cell type with Metascape (https://metascape.org/). The marker genes or signature genes for cell types were as follows:^[^ ^11^, ^17^, ^31^, ^32^, ^33^ ^]^ epithelial cells (EPCAM, KRT5, KRT8 and CDH1), B cells (CD19, MS4A1, CD79A, CD79B, BLNK), endothelial cells (CDH5,PECAM1,VWF, ENG,SELE), neutrophils (CD55, PCGR2A), nature killer cells (KLRB1, KLRC1, KLRD1, KLRF1, KLRK1, NCAM1), T cells (CD2, CD3D, CD3E, CD3G), macrophages (APOE, C1QA, C1QB), fibroblasts (DCN, TNFAIP6, APOD, FBLN1, FGF2, PTGDS, RSPO3, MYH11, GJA4, RGS5, MT1A), and undefined cells (STMN1, HMGB2, TYMS, UBE2C, PCLAF).

### Inference of Copy Number and Identification of Cancer Cells and Subtypes

Copy number analysis was performed with the R package inferCNV (v1.0.6) under default parameters. To identify tumor cells from epithelial cells, the authors followed the recently published approach by Ashley Maynard et al.^[^ ^34^ ^]^ Briefly, 500 non‐malignant cells, including macrophages, endothelial cells, and T cells, were randomly selected as a normal reference, and another 300 non‐malignant cells (as spike‐in cells) were randomly selected together with epithelial cells for CNV inference and hierarchical clustering as described. Then, the dendrogram was cut at the highest point at which all the spike‐in controls belonged to one cluster. The epithelial cells that clustered together with normal spike‐in controls were identified as “normal epithelial cells”, whereas the remaining cells were identified as “tumor cells”. Then, tumor cells were run with inferCNV again to infer CNV and tumor subclones. The tumor cells were re‐clustered with the Seurat package (resolution = 0.12), each subcluster was defined as a transcriptional subtype. The marker genes or signature genes for cell types or subtypes were referred:^[^ ^11^, ^17^, ^32^, ^33^ ^]^ epithelial cells (EPCAM, KRT5, KRT8, CDH1), luminal A cells (MSMB, KLK2, KLK3, NPY), luminal B cells (SLC14A1, MEG3, FHL2, KRT23), luminal C cells (TACSTD2, PSCA, KRT4, PIGR), basal cells (KRT5, KRT15, TP63), EMT genes (TWIST1, CDH2, CDH11, FN1, VIM, SNAI1, ZEB1, ZEB2, DCN), and stemness genes (ALDH1A1, CD44, PROM1, NANOG, KIT, NES, KLF4, CD55, ALCAM, NOTCH4, WNT7A, PDPN).

### Developmental Pseudotime Analysis

All tumor cell expression data were used for single‐cell pseudotime analysis using the Monocle (v2.16.0) package^[^ ^35^ ^]^ under default parameters. Highly variable genes were selected by the built‐in DispersionTable function of Monocle.

### Cell Cycle Analysis

Based on the 43 G1S genes and 54 G2M genes defined in the previous study,^[^ ^36^ ^]^ the authors calculated the G1S scores and G2M scores for each malignant tumor cell with the CellCycleScoring function in Seurat. They defined a cell as cycle‐active as long as one score was greater than 0.1.

### Transcription Factor Analysis

To explore the dominant transcription factor in different subclones, the authors applied SCENIC with the SCENIC package (v1.2.1)^[^ ^37^ ^]^ in R. First, they inferred potential targets of each TF based on their co‐expression function indicated by GENIE3. Then, potential direct binding targets were selected according to DNA motif analysis and compared with the reference motif database downloaded from RcisTarget (https://resources.aertslab.org/cistarget/). The regulon activity of each cell was analyzed via the AUCell function. Each cell was scored for regulon activity using the function AddModuleScore in Seurat, and final visualization was implemented with the featurePlot function in Seurat.

### Cell‐Cell Communication Analysis

To enable a comprehensive analysis of cell–cell communication molecules, the authors applied cell–cell communication analysis based on CellChat (v1.0.0).^[^ ^18^ ^]^ The ligand and receptor genes expressed by each cell were projected into a manually selected reference communication network and the probability of communication in each pathway was inferred by gene expression. They statistically analyzed the communication probability between subclones and other non‐malignant cells as well as the communication between different subclones. Histograms were implemented with the ggplot2 package (v3.3.2) in R.

### Information Entropy of Transcriptional Subtypes and the Tumor Ecosystem

An online tool^[^ ^38^ ^]^ (https://cibersortx.stanford.edu/) was used to construct the cell‐type‐specific signature matrix according to the top 200 marker genes of each cell type, and then the constructed signature matrix was used to infer the fractions of different cell types and transcriptional subtypes. Bulk expression data from the TCGA PRAD project were downloaded from the GDC data portal (https://portal.gdc.cancer.gov/) and all 495 patients were included in the analysis. For each patient, the information entropy contained in its expression data was calculated by using the following formula:

(1) $$H\;\left(X\right)=\;-\sum_{i\;\in \;X}p\left(i\right)*\log\left(p\left(i\right)\right)$$

where *p* represents the fraction of cell type *i* in the patient, and *X* represents the collection of all cell types or transcriptional subtypes.

Notably, to calculate the information entropy of the ecosystem, the authors calculated the fraction of all epithelial cells (malignant and non‐malignant) as a whole; to calculate the information entropy of the transcriptional subtype, the fraction of each transcriptional subtype in all tumor cells was calculated. Then, the correlation analysis of information entropy between transcriptional subtypes and the ecosystem was performed in R (v4.0.3), and further visualization was implemented with the pheatmap (v1.0.12) and ggplot2 (v3.3.2) packages.

### Clinical Correlation Analysis in TCGA RNA‐seq Data

As described above, the authors obtained the fractions of various cell types in the tumor ecosystem of patients in the PRAD project through the CIBERSORTx online tool.^[^ ^38^ ^]^ All 495 patients were divided into two groups by hierarchical clustering for the proportion of all cell types according to the results of CIBERSORTx or by the median cell proportion for each cell type. Progression‐free survival (PFS) was used as a clinical endpoint, and the KM curve was plotted using the survminer (v0.4.8) package.

### Immunohistochemistry (IHC)

Primary antibodies included ZEB1(21544‐1‐AP, Proteintech, 1:50), KLK3(ZM‐0218, ZSGB‐BIO), TACSTD2(ab214488, Abcam, 1:500), and KRT15(ab52816, Abcam, 1:200). Formalin‐fixed and paraffin‐embedded tissue sections (5 µm) were deparaffinized and rehydrated. Antigen retrieval was carried out using 10 mm sodium citrate (pH 6.0). Endogenous peroxidase activity was blocked with 0.3% hydrogen peroxide for10 min and 5% BSA in PBS for 1 h. Slides were incubated overnight at 4 °C with a primary antibody, which was followed by incubation with an HRP‐linked secondary antibody (PV‐9001, ORIGENE) at room temperature (30 min). Diaminobenzidine (DAB) was used as chromogen, and the sections were counterstained with haematoxylin. Representative fields were selected from these slides.

### Data Availability

Data have been deposited in the Genome Sequence Archive for Human (GSA‐Human) under accession HRA000823^[^ ^39^, ^40^ ^]^ (https://ngdc.cncb.ac.cn/gsa‐human/s/Bv7gBES8). For survival analysis, bulk RNA‐seq data from the following studies were used: TCGA (495 samples, http://firebrowse. org/?cohort = PRAD).^[^ ^41^, ^42^ ^]^ All other data supporting the findings of this study are available from the corresponding author on reasonable request.

### Statistical Analysis

All statistical analyses of scRNA‐seq data were performed using the Cell Ranger software, Seurat, SingleR, Metascape website, inferCNV, Monocle, SCENIC, CellChat, CIBERSORTx online tool, survival, survminer, and GSVA. Visualization of data was made using R packages ggplot2 and pheatmap. The data in Figures 3I, 5A,B, and 6E were presented as median with interquartile range (IQR). The chi‐squared test was used to reveal the statistical difference in Figure 3D and two‐tailed *t*‐test was used to reveal the statistical difference in Figures 3I and 6E. Correlations were identified by Pearson's or Spearman's correlation test in Figure 6C, and Figures S4B and S6B, Supporting Information. Log‐rank test was performed in Kaplan–Meier survival analysis in Figure 6 and Figure S6, Supporting Information. A *p*‐value or *q*‐value less than 0.05 was considered as significant difference. All statistical analyses were conducted in R4.0.3.

## Conflict of Interest

The authors declare no conflict of interest.

## Author Contributions

G.G., Y.H., J.Z., and X.L. contributed equally to this work. W.C., H.H., L.Z., X.L. conceived the study. G.G., Y.H., J.Z., and X.L. designed the experiments. G.G., Y.H., J.Z., X.L., X.L., Y.G., Z.L., J.W., W.Z., Y.X., Y.P., J.D., and B.Z. performed the experiments and analyzed the data. W.C., G.G., Y.H., and J.Z. wrote the paper.

## Supporting information

Supporting Information

Supplemental Video 1

Supplemental Video 2

Supplemental Video 3

## Data Availability

The data that support the findings of this study are available from the corresponding author upon reasonable request.
