# Supplementary material for: Single‐Cell RNA‐seq Reveals a Developmental Hierarchy Super‐Imposed Over Subclonal Evolution in the Cellular Ecosystem of Prostate Cancer
Source: Adv Sci (Weinh). 2022 Mar 24;9(15):2105530. doi: 10.1002/advs.202105530 (PMC9131431; doi:10.1002/advs.202105530)
Supplement: Supplementary file 1 — Supporting Information [file ADVS-9-2105530-s002.pdf]

ADVANCED SCIENCE
Open Access

Supporting Information

for *Adv. Sci.*, DOI 10.1002/advs.202105530

Single-Cell RNA-seq Reveals a Developmental Hierarchy Super-Imposed Over Subclonal Evolution in the Cellular Ecosystem of Prostate Cancer

Guangzhe Ge, Yang Han, Jianye Zhang, Xinxin Li, Xiaodan Liu, Yanqing Gong, Zhentao Lei, Jie Wang, Weijie Zhu, Yangyang Xu, Yiji Peng, Jianhua Deng, Bao Zhang, Xuesong Li, Liqun Zhou*, Huiying He* and Weimin Ci**

Supporting Information

Single-cell RNA-seq reveals a developmental hierarchy super-imposed over subclonal evolution in the cellular ecosystem of prostate cancer

Guangzhe Ge[a,1], Yang Han[a,c,1], Jianye Zhang[a,d,1], Xinxin Li[a,1], Xiaodan Liu[b], Yanqing Gong[d,e,f], Zhentao Lei[g], Jie Wang[d,e,f], Weijie Zhu [d,e,f], Yangyang Xu[d,e,f], Yiji Peng[d,e,f], Jianhua Deng[h], Bao Zhang[g], Xuesong Li[d,e,f,]*, Liqun Zhou[d,e,f,]*, Huiying He[b,]*, Weimin Ci [a,c,i]*

*Corresponding author:

Weimin Ci (ciwm@big.ac.cn);

Huiying He (huiyinghe@bjmu.edu.cn);

Liqun Zhou (zhoulqmail@sina.com);

Xuesong Li(pineneedle@sina.com);

[1] These authors contributed equally to this work.

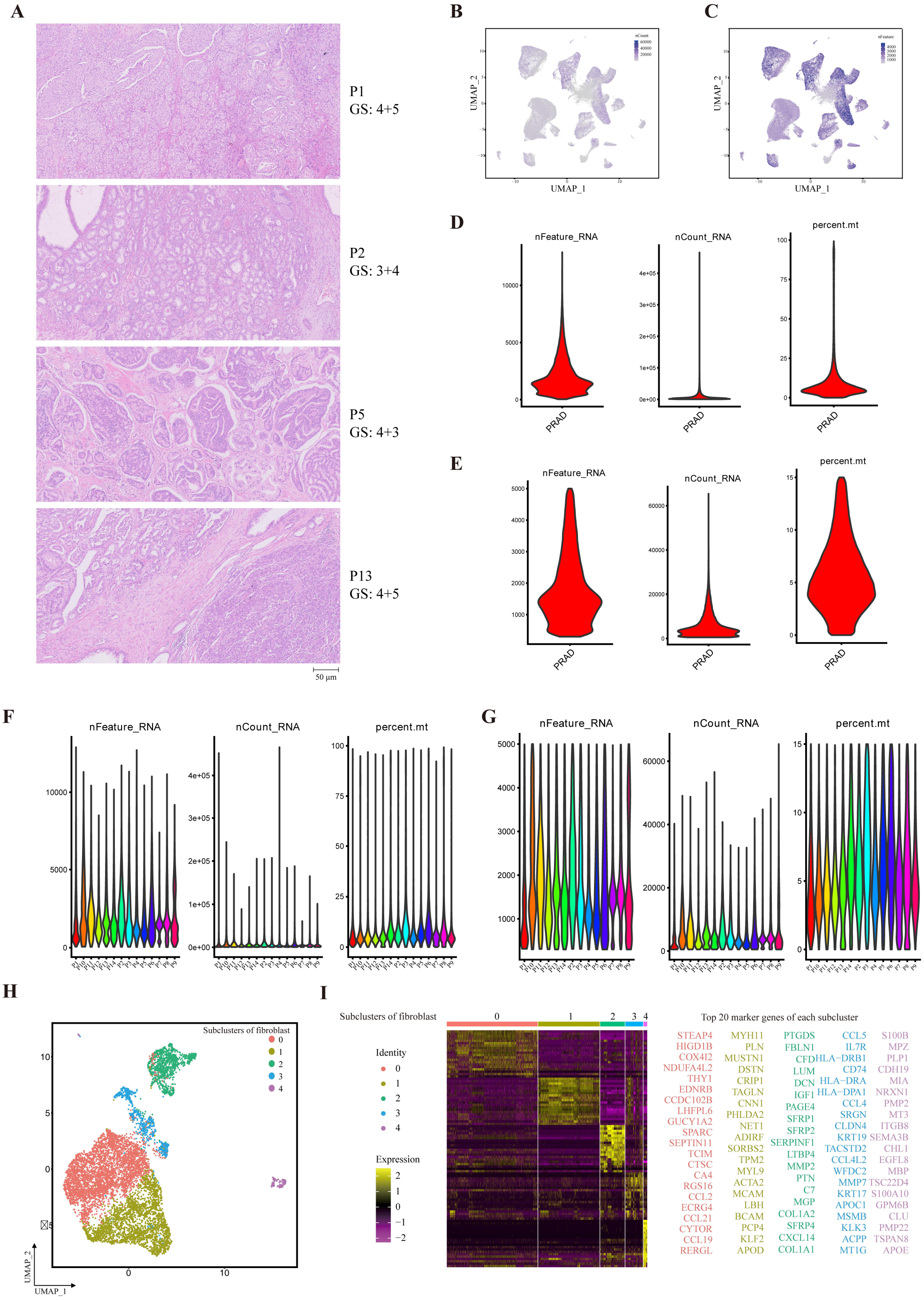

Fig. S1-Quality control of our prostate cancer scRNA-seq dataset. (A) Haematoxylin and eosin (H&E) staining for 4 representative prostate cancer patients. The GS represents Gleason_score.

(B, C) The number of transcripts (B) and detected genes (C) in each cell among 14 patients. (D, F) The data quality for all cells (D) or across different patients (F) before filtering. (E, G) The data quality for all cells (E) or across different patients (G) after filtering. (H) UMAP visualization of different fibroblasts subtypes across 14 patients by unsupervised clustering. (I) The top 20 marker genes for each fibroblasts subtypes were shown.

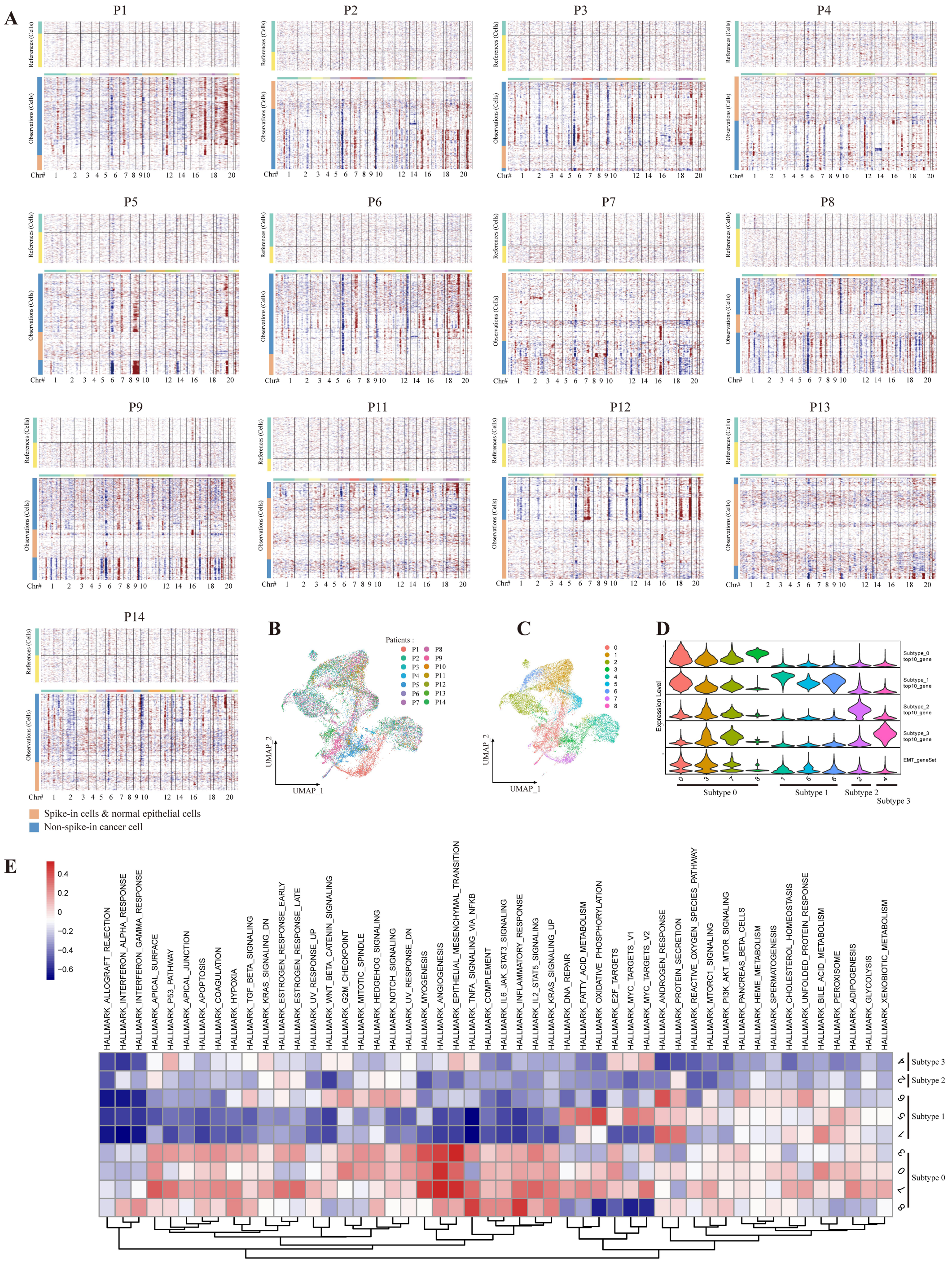

Fig. S2-The tumour cells and subclones are identified by inferCNV in other 13 patients. (A) The tumour cells and normal epithelial cells are identified by inferCNV in 13 patients (left). (B) Tumour cells were colored by patient ID in UMAP. (C)

Tumor cells were re-clustered into 9 subpopulations by unsupervised clustering. (D) Different geneset score were calculated and visualized in 9 tumor subpopulations. (E) Differences among 9 tumor subpopulations in 50 hallmark pathway activities were scored with GSVA software.

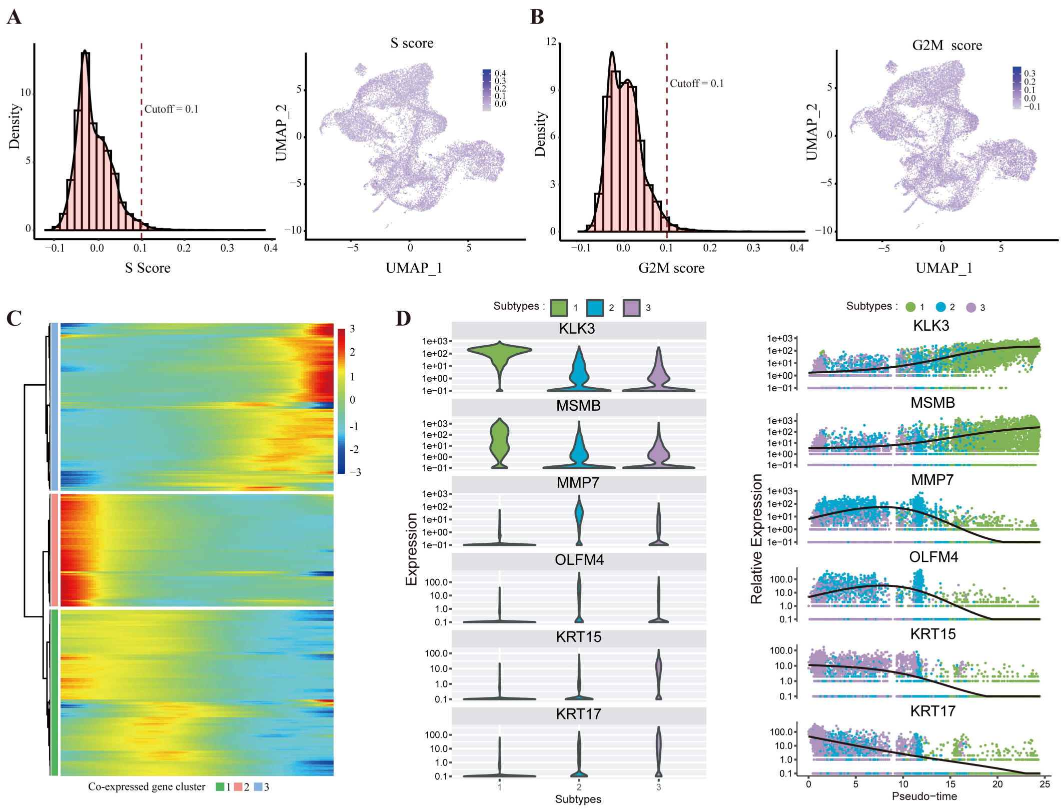

Fig. S3-Heterogeneity of the cell cycle and developmental hierarchy in tumour cells. (A) Distributions of the S score in tumour cells. (B) Distributions of the G2M score in tumour cells. (C) Monocle analysis of 4 tumour subtypes. (C) Heatmap shows pseudotime-associated DEGs in the epithelial-associated tumour subtypes. Cells are ordered by pseudotime. (D) The expression changes in cluster genes positively and negatively correlated with the tumour subtypes (left) and pseudotime (right).

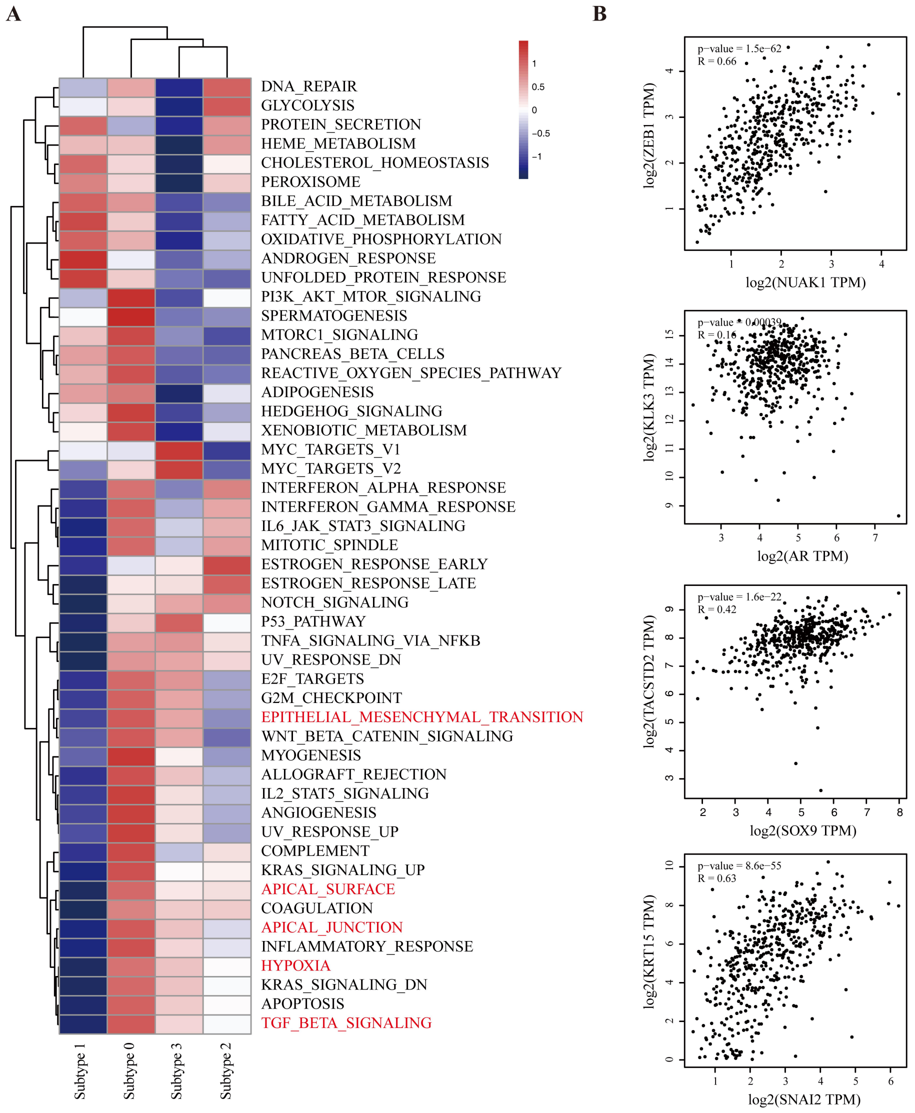

Fig. S4-The prediction of TF regulons that govern different tumour subtypes. (A) Differences in 50 hallmark pathway activities scored with GSVA software. Shown are t values calculated by a linear model.（B）The Spearman’s correlation coefficients of gene expression are shown in the TCGA cohort of prostate cancer patients.

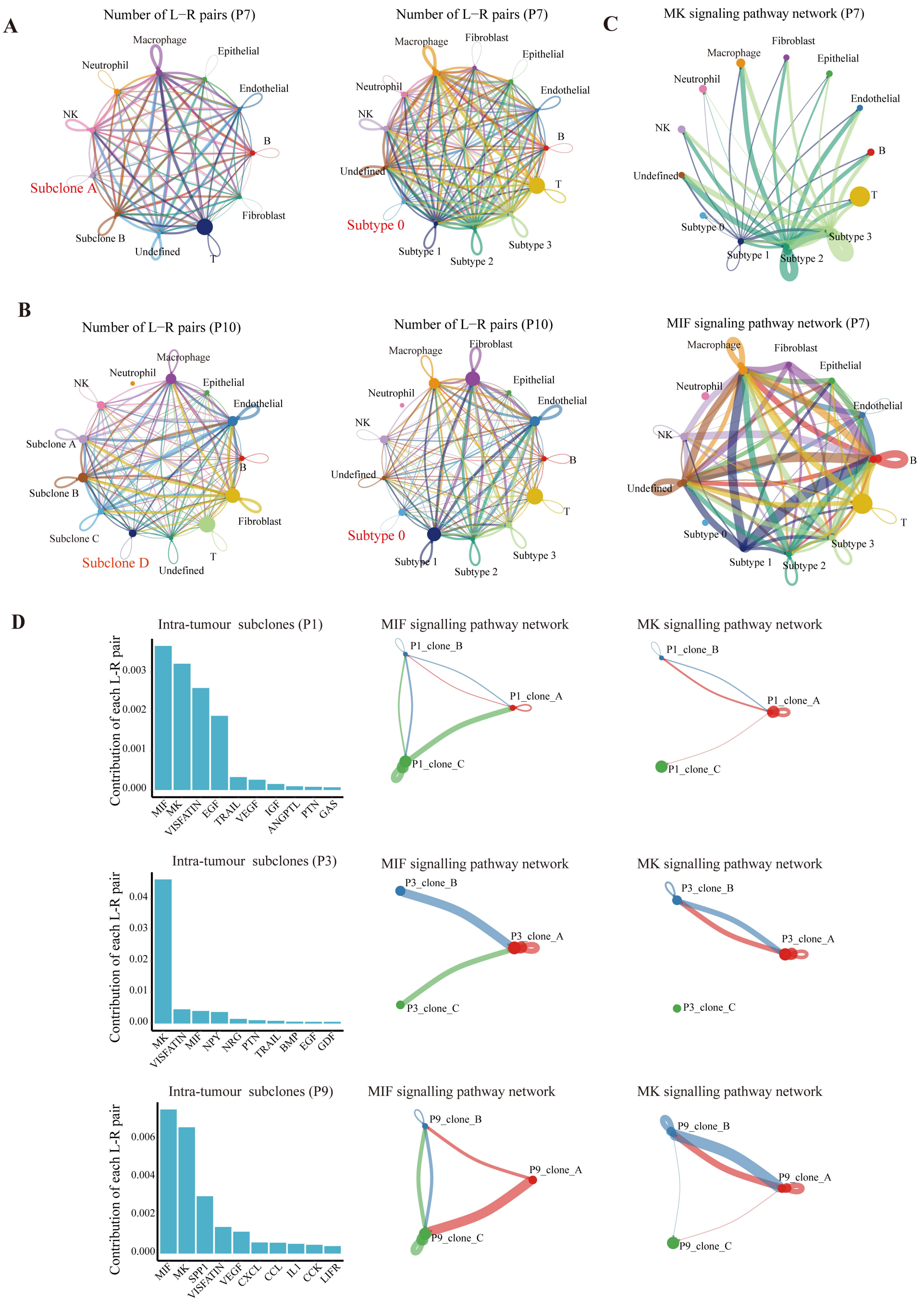

Fig. S5-Differential strengths of cell communication exist within different tumour subclones and cell types in the tumour microenvironment. (A) and (B) Number of significant ligand-receptor pairs between any pair of two cell populations. The edge width is proportional to the indicated number of ligand-receptor pairs. Circle sizes are proportional to the number of cells in each cell group and edge width represents the

communication probability. The subclone with dominant subtype 0 phenotype highlighted in red. (C) The inferred MK and MIF signaling networks in one representative patient, P7. (D) The difference in cell communication strength between different subclones was calculated in P1, P3 and P9. The signaling networks were shown within subclones in MK and MIF pathways.

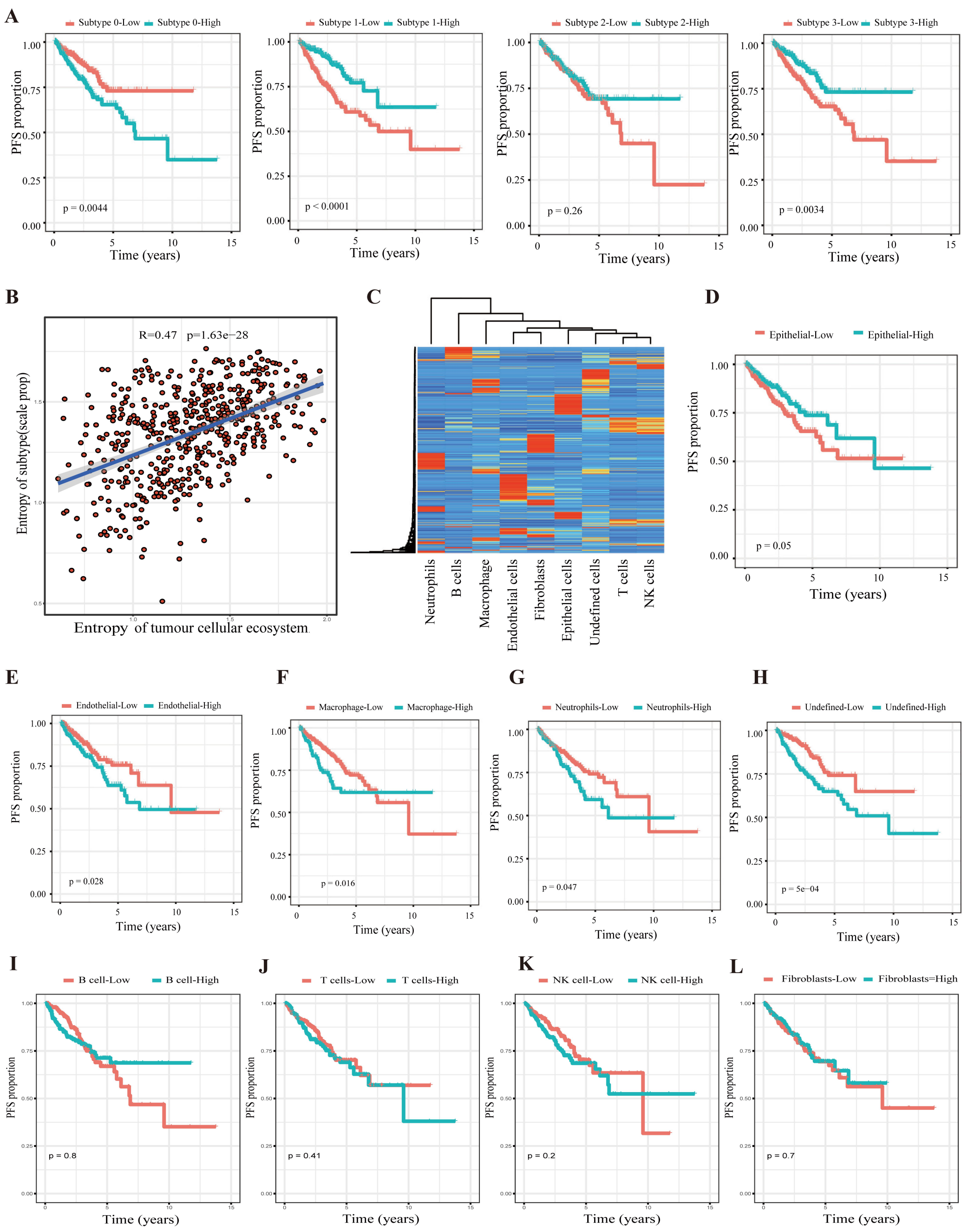

Fig. S6-The "tumour ecosystem subtype" defined by cell type composition in the tumour microenvironment predicts patient prognosis in the TCGA cohort. (A) Kaplan-Meier plot shows that the Subtype 0-High group, based on its median cell fraction, was associated with shorter PFS than the Subtype 1/2/3-High group in the TCGA dataset. (B) Pearson correlation coefficient of tumour cell transcriptional

heterogeneity within 9 tumor subpopulations and cellular ecosystem diversity. Shannon's diversity index (SHDI) is used to evaluate heterogeneity. (C) The signature gene matrix used to deconvolute the TCGA dataset was calculated by CIBERSORTx using our scRNA-seq data. (D-L) Kaplan-Meier plot of the relationship between the cell content-high and cell content-low groups in epithelial cells (D), endothelial cells (E), macrophages (F), neutrophils (G), undefined cells (H), B cells (I), T cells (J), NK cells (K) and fibroblasts (L) within the TCGA dataset. P value was calculated by log-rank test.

Supplementary Table 1. Basic information for patients.

Supplementary Table 2. Marker genes for different cell types in tumor microenvironment.

Supplementary Table 3. Marker genes for different tumor subtypes in prostate cancer patients.
